# Outpatient diuretic intensification: a simple prognostic marker in cardiac transthyretin amyloidosis

**DOI:** 10.1007/s00392-025-02617-4

**Published:** 2025-03-04

**Authors:** Richard J. Nies, Svenja Ney, Jasper F. Nies, Katharina Seuthe, Lukas Klösges, Monique Brüwer, Stephan Nienaber, Sascha Macherey-Meyer, Matthieu Schäfer, Roman Pfister

**Affiliations:** 1 Clinic III for Internal Medicine, Faculty of Medicine and University Hospital Cologne, University of Cologne, Kerpener Str. 62, 50937 Cologne, Germany; 2 Department of Nephrology, University of Cologne, Kerpener Str. 62, 50937 Cologne, Germany

**Keywords:** Cardiac transthyretin amyloidosis, Risk stratification, Prognosis, Outpatient diuretic intensification, Cardiomyopathy

## Abstract

**Background:**

Currently, simple clinical parameters indicating disease progression are lacking in patients with transthyretin amyloid cardiomyopathy (ATTR-CM). This study aimed to evaluate the prognostic value of outpatient diuretic intensification (ODI) in ATTR-CM patients.

**Methods:**

This retrospective study examined ATTR-CM patients at a tertiary care center between August 1, 2020, and June 30, 2023. ODI was defined as any loop diuretic increase within 6 months after baseline visit, and its impact on all-cause mortality and hospitalization for heart failure (HF) was analyzed.

**Results:**

Altogether, 182 patients were included (median age 80 [76; 84] years; 88% male), and 25% experienced ODI (median increase 10 [10; 40] mg furosemide equivalent). Independent predictors of ODI were higher baseline New York Heart Association (NYHA) class and polyneuropathy. Both any ODI and the magnitude of furosemide equivalent increase were significantly associated with mortality and HF hospitalization during a median follow-up of 17 months. After adjusting for baseline NYHA class and National Amyloidosis Centre stage, significantly increased risk of all-cause mortality (hazard ratio [HR] 2.38, 95% confidence interval [CI] 1.03–5.53; *p* = 0.043) and HF hospitalization (HR 3.27, 95% CI 1.41–7.60; *p *= 0.006) persisted in patients with ODI. Its prognostic value was similar in strata of age, ATTR subtype, previous cardiac decompensation, biomarkers, left ventricular ejection fraction, six-minute walk distance, and tafamidis treatment.

**Conclusion:**

ODI occurred in one in four ATTR-CM patients within 6 months and was associated with more severe baseline amyloid organ manifestations. ODI and the magnitude of diuretic dose increase provide easily assessable clinical markers of disease progression in patient monitoring.

**Graphical abstract:**

A total of 182 patients diagnosed with transthyretin amyloidosis cardiomyopathy (ATTR-CM) were analyzed for an increase in loop diuretic dosage within the first 6 months after the baseline visit. Twenty-five percent of the cohort experienced outpatient diuretic intensification (ODI), with independent predictors being dyspnea in higher New York Heart Association (NYHA) class and polyneuropathy (PNP). ODI was significantly associated with all-cause mortality, and its prognostic value remained consistent across various risk factors

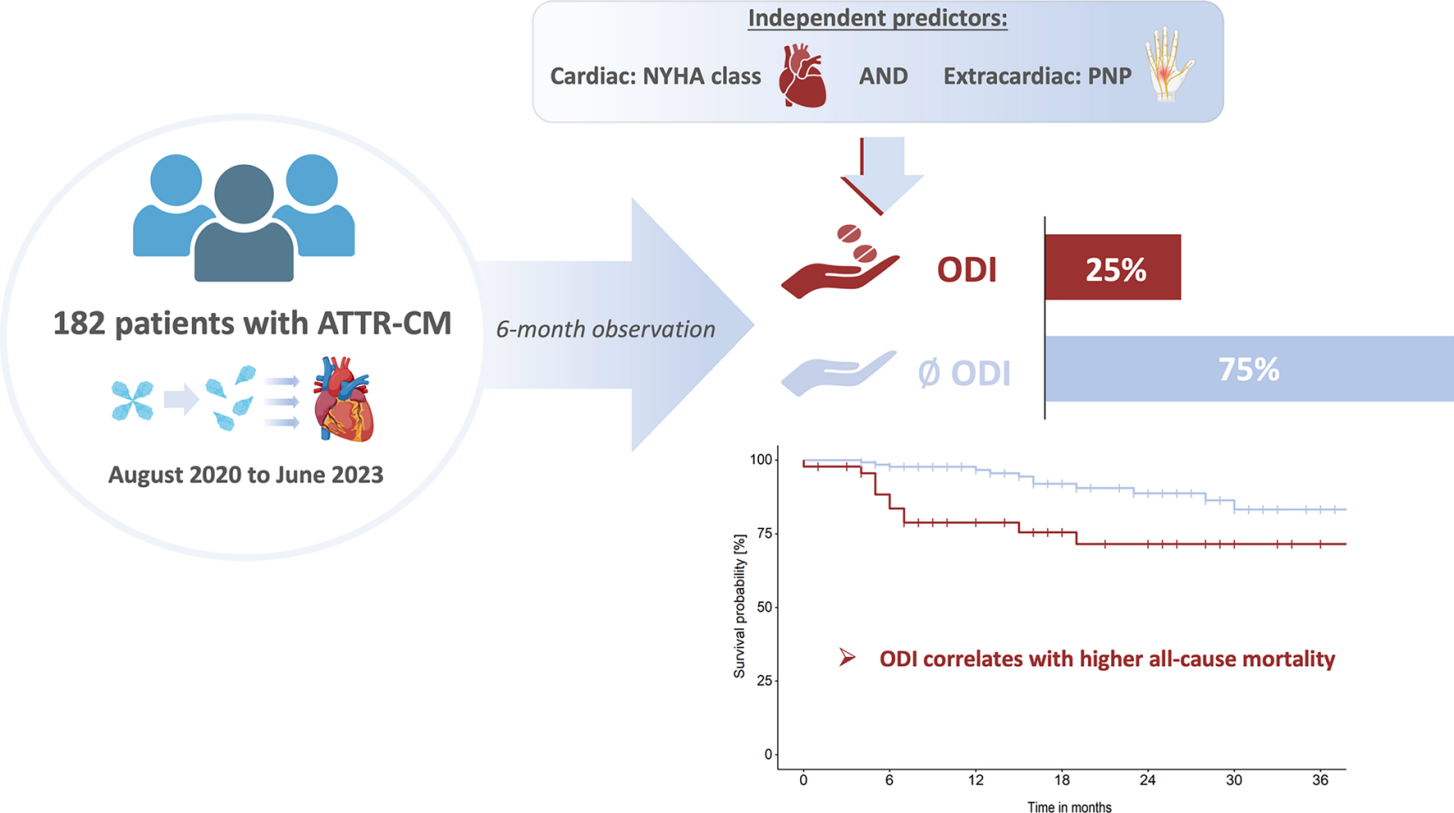

**Supplementary Information:**

The online version contains supplementary material available at 10.1007/s00392-025-02617-4.

## Introduction

Transthyretin amyloid cardiomyopathy (ATTR-CM) is a progressive disease characterized by the extracellular deposition of misfolded transthyretin amyloid fibrils [1]. Once considered a rare disease, ATTR-CM is a relevant cause of heart failure (HF) comprising more than 10% in elderly patients with myocardial thickening and preserved ejection fraction [2, 3].

The development of drugs with disease-modifying effects in ATTR-CM [4, 5] prompted the need to refine risk stratification and monitoring of disease progression in patients with ATTR-CM as a basis for individualized management and treatment decisions. An expert consensus statement of the European Society of Cardiology (ESC) on monitoring of ATTR-CM recommends routine assessments of clinical parameters, biomarkers, and cardiac imaging reflecting HF severity [6, 7]. However, cut-offs of such parameters defining disease progression are unclear and prognostic impact remains uncertain [8]. Even more important from the practical point of view, serial cardiac imaging requires significant time and resources, and interpreting cardiac biomarkers such as N-terminal pro-brain natriuretic peptide (NTproBNP) can be complex due to interference with comorbidities [7, 9, 10]. Therefore, there is a need for easily assessable clinical parameters to monitor disease progression and related risk in ATTR-CM.

Outpatient diuretic intensification (ODI) due to signs of congestion has recently gained much attention in the context of the “worsening HF” concept [11]. Importantly, ODI is more common than HF hospitalizations and is independently associated with cardiovascular death and hospitalization in general HF patients [12, 13]. The purpose of this study was to investigate the frequency, predictors, and clinical significance of ODI in contemporary patients with ATTR-CM. We hypothesized that ODI could provide significant prognostic value to treating physicians.

## Methods

### Study population

In this single-center, retrospective observational study, we included consecutive patients who presented to the cardiology department of a tertiary care center for the first time with ATTR-CM between August 1, 2020, and June 30, 2023. The first diagnosis of cardiac amyloidosis could have been made before introduction to our center, but the date of the initial visit to our department was considered as baseline. The diagnosis of ATTR-CM was made in accordance to the recommendations of the ESC cardiac amyloidosis working group and the 2023 ESC Guidelines for the Management of Cardiomyopathies [14, 15].

Exclusion criteria were missing data on the exposure variable (loop diuretic dose at baseline or during the first six months after the initial visit), as well as death within the exposure period of the first 6 months (Online Resource 1). All analyses were performed using anonymized data obtained with informed consent from all participants.

### Data collection

Clinical baseline and follow-up data, including demographics, comorbidities, biomarkers, concomitant medication, imaging and other diagnostic results, were systematically collected from routine assessments. The United Kingdom National Amyloidosis Centre (UK-NAC) and the Mayo risk models for ATTR-CM were calculated as reported [7, 16]. Transthoracic echocardiography (TTE) was performed according to the current recommendations of the American Society of Echocardiography [17, 18]. The six-minute walk distance (6-MWD) was used for functional assessment [19, 20]. The exposure variable ODI was defined by any temporary or sustained increase of loop diuretic dosage within the first 6 months after the baseline visit. The decision for ODI was made by the treating physician solely based on clinical signs of volume overload, i.e., peripheral edema or progressive dyspnea with pulmonary rales or pleural effusion. The dosage of loop diuretics was assessed as furosemide equivalents at baseline and monitored over the observational period. Oral torasemide dose was converted at a ratio of 2:1 to furosemide [21]. Information on hospitalization and vital status was gathered from clinical records, telephone interviews, and/or reports from primary physicians.

### Clinical outcomes

The primary endpoint of the study was all-cause mortality. The key secondary endpoint was hospitalization for HF, which was defined as inpatient admission due to cardiac decompensation with fluid overload as assessed and reported by treating physicians.

### Data analysis

Data are presented as count (percent), mean (standard deviation, SD), or median (quartiles) as appropriate. The study cohort was analyzed by ODI within the first 6 months after initial presentation. Characteristics of patients with and without ODI were compared using the unpaired *t* test, Mann–Whitney *U* test, or Chi-square test as appropriate. Normal distribution was tested using the Kolmogorov–Smirnov or Shapiro–Wilk tests.

Odds ratios (OR) and their corresponding 95% confidence intervals (CI) for ODI were calculated using univariate logistic regression analysis for all variables significantly associated with ODI. A multivariate logistic regression model was calculated by including NYHA class as the most important clinical HF parameter in the first block and using a backward likelihood ratio elimination for all additional variables tested univariately in the second block. The impact of ODI on all-cause mortality and hospitalization due to HF was assessed using Kaplan–Meier curves and the log-rank test. Univariate and multivariate Cox regression analyses were employed to calculate the hazard ratio (HR) and corresponding 95% CIs. In adjusted regression analysis, pre-specified clinically and prognostically relevant variables were included (NYHA class and UK-NAC stage). Observational time in survival and hospitalization analysis started 6 months after the baseline visit to account for the exposure to “ODI during the first six months after baseline”. Forest plots depicted the corresponding HRs and CIs for pre-specified subgroups, which were predominately based on risk stratification variables of HF. Subgroups with fewer than two events were not analyzed. A two-sided *p* value of < 0.05 was considered statistically significant in all tests. Statistical analyses were performed using IBM SPSS Statistics, version 29.0.0.0. Kaplan–Meier survival plots were generated in R (version 4.2.0) with the UpSetR (version 1.4.0), survival (version 3.3-1), and survminer (version 0.4.9) packages. Forest plots were created using Microsoft Excel for Mac (version 16.86) and Microsoft PowerPoint (version 16.86) for Mac.

## Results

### Study population

The final study cohort comprised 182 patients with ATTR-CM. The median age was 80 years (interquartile range [IQR]: 76; 84), 88% (160/182) were male, and 7% (13/182) were diagnosed with variant (v) ATTR-CM (Table 1). Polyneuropathy (PNP), CTS, and lumbar spinal stenosis were identified in 54% (99/182), 45% (81/182), and 14% (26/182) of the patients from history and records, respectively. Nearly two-thirds (117/182) of the total cohort and 86% (85/99) of patients with a diagnosis of PNP had undergone a specialized neurological examination confirming the validity of diagnosis. Overall, 43% (78/182) of the patients were classified with dyspnea in NYHA class III or IV, and 20% (36/182) had a history of hospitalization due to HF. The mean 6-MWD at the initial visit was 343 ± 114 m. According to the UK-NAC staging system, 56% (102/182) of patients were in stage I, 31% (56/182) in stage II, and 13% (24/182) in stage III [7]. According to the Mayo risk model, the corresponding rates were 44% (62/141), 33% (47/141), and 23% (32/141) [16]. The median interventricular septal thickness in diastole (IVSd) was 17 mm (IQR: 15; 19) and median average global longitudinal strain (GLS) was −11.0% (IQR: −14; −8.8).

**Table 1:** Baseline characteristics of the study cohort and by ODI

	ATTR-CM cohort	No ODI within 6 months	ODI within 6 months	*p*
	*n* = 182	*n* = 136 (74.7%)	*n* = 46 (25.3%)	
Type of ATTR-CM				
Wild-type; %	55.5	56.6	52.2	
Variant; %	7.1	6.6	8.7	0.826
Not tested; %	37.4	36.8	39.1	
Time since first diagnosis (months); Mdn [Q_1_;Q_3_]	2 [1; 3]	2 [1; 3]	2 [0; 5]	0.906
Demographic data				
Male patients; %	87.9	89	84.8	0.451
Age (years); Mdn [Q_1_; Q_3_]	80 [76; 84]	80 [75; 83]	82 [78; 85]	0.067
BMI (kg/m^2^); M ± SD	25.8 ± 3.3	25.9 ± 3.3	25.6 ± 3.1	0.619
Modified BMI [(kg/m^2^)x(g/l)]; M ± SD	1111 ± 153	1118 ± 154	1088 ± 146	0.256
Vital parameters				
SBP (mmHg); M ± SD	137 ± 21 (*n* = 152)	136 ± 21 (*n* = 110)	138 ± 21 (*n* = 42)	0.586
DBP (mmHg); M ± SD	78 ± 11 (*n* = 152)	78 ± 11 (*n* = 110)	79 ± 11 (*n* = 42)	0.413
Heart rate (1/min); M ± SD	74 ± 14 (*n* = 175)	73 ± 13 (*n* = 130)	78 ± 15 (*n* = 45)	0.034
Symptoms				
NYHA class				
I; %	8.2	8.8	6.5	
II; %	48.9	55.9	28.3	0.005
III; %	41.8	34.6	63	
IV; %	1.1	0.7	2.2	
History of cardiac decompensation; %	19.8	19.9	19.6	0.966
Comorbidities				
History of arterial hypertension; %	76.4	77.2	73.9	0.649
Diabetes mellitus; %	15.4	13.2	21.7	0.167
CAD; %	40.1	39	43.5	0.59
AF; %	57.1	53.7	67.4	0.104
History of CTS; %	44.5	45.6	41.3	0.613
Lumbar spinal stenosis; %	14.3	9.6	28.3	0.002
History of SAVR or TAVI; %	8.8	8.8	8.7	0.979
PNP; %	54.4	48.5	71.7	0.006
PM; %	16.5	18.4	10.9	0.235
History of stroke, %	7.1	7.4	6.5	0.85
Medications				
Furosemide equivalent dose (mg); Mdn [Q_1_; Q_3_]	10 [0; 20]	10 [0; 20]	10 [0; 30]	0.203
0 mg; %	41.2	43.4	34.8	
1–20 mg; %	38.5	39	37	
21–40 mg; %	12.1	11	15.2	0.474
41–80 mg; %	6	4.4	10.9	
> 80 mg; %	2.2	2.2	2.2	
Non-loop diuretics; %	51.1	50.7	52.2	0.866
Thiazide; %	20.9	19.1	26.1	0.315
MRA; %	26.9	27.2	26.1	0.882
SGLT2i; %	13.7	14.7	10.9	0.513
ACEi, AT1-I or ARNI; %	74.2	72.8	78.3	0.464
Beta-blocker; %	58.2	58.1	58.7	0.942
Oral anticoagulant; %	62.1	59.6	69.6	0.227
Biomarkers				
Serum albumin (g/l); Mdn [Q_1_; Q_3_]	43 [41; 45]	44 [41; 45]	42 [41; 44]	0.094
NTproBNP (pg/ml); Mdn [Q_1_; Q_3_]	2099 [998; 3837]	1716 [857; 3268]	2703 [1588; 4831]	0.002
GFR (ml/min); M ± SD	58 ± 19	58.9 ± 18.9	54.6 ± 18.2	0.181
Troponin T (ng/ml); Mdn [Q_1_; Q_3_]	0.047 [0.035; 0.070] (*n* = 141)	0.045 [0.032; 0.065] (*n* = 104)	0.060 [0.039; 0.089] (*n* = 37)	0.016
UK-NAC stage				
I; %	55.5	58.8	45.7	0.12
II or III; %	44.5	41.2	54.3	
Mayo stage				
I; %	44.0 (*n* = 141)	48.1 (*n* = 104)	32.4 (*n* = 37)	0.1
II or III; %	56.0 (*n* = 141)	51.9 (*n* = 104)	67.6 (*n* = 37)	
Imaging				
LVEF (%); M ± SD	57 ± 8	57 ± 7	55 ± 9	0.334
IVSd (mm); Mdn [Q_1_; Q_3_]	17 [15; 19] (*n* = 179)	17 [15; 18] (*n* = 135)	18 [16; 20] (*n* = 44)	0.029
E/e’; M ± SD	15.1 ± 7.1 (*n* = 114)	14.3 ± 6.3 (*n* = 87)	17.8 ± 8.7 (*n* = 27)	0.023
GLS (%); Mdn [Q_1_; Q_3_]	−11.1 [−14.2; −8.8] (*n* = 168)	−11.3 [−14.0; −9.1] (*n* = 125)	−9.5 [−15.0; −7.7] (*n* = 43)	0.054
sPAP (mmHg); M ± SD	36.3 ± 13.4 (*n* = 135)	38.0 ± 13.7 (*n* = 97)	42.6 ± 12.0 (*n* = 38)	0.077
TAPSE (mm); M ± SD	17.8 ± 4.9 (*n* = 177)	18.2 ± 5.1 (*n* = 134)	16.7 ± 4.1 (*n* = 43)	0.082
Pericardial effusion; %	10.9	8.8	17.4	0.108
6-MWD (m); M ± SD	343 ± 114 (*n* = 116)	358 ± 117 (*n* = 87)	295 ± 91 (*n* = 29)	0.009
ATTR specific medication				
Tafamidis 61 mg; %	84.1	83.8	84.8	0.878
Inotersen or Patisiran; %	1.6	2.2	0	0.31

*Of 142 patients in whom tafamidis 61 mg was initiated, treatment was discontinued in 15 cases and switched to Vutrisiran in two cases (vATTR). The reasons for discontinuation were disease progression (*n* = 9) or side effects (*n* = 2). In four patients, the reasons for tafamidis discontinuation remained unclear

*6-MWD* six-minute walk distance, *ACEi* angiotensin-converting enzyme inhibitor, *AF* atrial fibrillation, *ARNI* angiotensin receptor neprilysin inhibitor, *AT1-I* angiotensin 1 inhibitor, *ATTR* transthyretin amyloidosis, *ATTR-CM* transthyretin amyloidosis cardiomyopathy, *BMI* body mass index, *CAD* coronary artery disease, *CTS* carpal tunnel syndrome, *DBP* diastolic blood pressure, *GFR* glomerular filtration rate, *GLS* global longitudinal strain, *IVSd* interventricular septal thickness in diastole, *LVEF* left ventricular ejection fraction, *M* mean, *Mdn* median, *MRA* mineralocorticoid receptor antagonist, *NTproBNP* N-terminal pro-brain natriuretic peptide, *NYHA* New York Heart Association, *ODI* outpatient diuretic intensification, *PM* pacemaker, *PNP* polyneuropathy, *Q* quartile, *RV* right ventricular, *SAVR* surgical aortic valve replacement, *SBP* systolic blood pressure, *SD* standard deviation, *SGLT2i* sodium/glucose cotransporter 2 inhibitor, *sPAP* systolic pulmonary arterial pressure, *TAPSE* tricuspid annular plane systolic excursion, *TAVI* transcatheter aortic valve implantation, *UK-NAC* United Kingdom National Amyloidosis Centre

Twenty-one percent (38/182) of patients received thiazides, 27% (49/182) mineralocorticoid receptor antagonists (MRA), and 14% (25/182) sodium-glucose cotransporter 2 inhibitor (SGLT2i). In total, 51% (93/182) of patients were taking one of the three non-loop diuretics. Angiotensin-converting enzyme inhibitors (ACEi), angiotensin II receptor type 1 inhibitors (AT1-I), or angiotensin receptor-neprilysin inhibitors (ARNI) were prescribed to 74% (135/182) of patients. Fifty-nine percent (107/182) of the cohort were on loop diuretics at the initial presentation, with a median furosemide equivalent dose of 20 mg (IQR: 10; 40). Tafamidis 61 mg was administered to 84% (153/182) of the patients, with treatment initiation in nearly all cases during the baseline visit. Gene silencers were prescribed in 1.6% (3/182) of the cohort. The median follow-up time starting 6 months after the baseline visit was 17 months (IQR: 9; 28). Follow-up information was complete in all patients.

### Outpatient intensification of oral loop diuretics

Within the first 6 months after the initial presentation, 25% (46/182) of patients experienced ODI. Among those who received ODI, the median absolute increase in furosemide equivalent dose was 10 mg (IQR: 10; 40), with 28% (13/46) experiencing an increase of more than 20 mg (Table 2). Demographic data and treatment with non-loop diuretics were not significantly different between groups with and without ODI. Patients in advanced disease stages (stage II or III according to both the UK-NAC and Mayo risk models) experienced ODI more frequently than those in early stages (stage I), though this difference was not statistically significant. However, patients with ODI were significantly more symptomatic according to NYHA class compared to patients without ODI.

**Table 2:** Absolute magnitude of increase in loop diuretic dose within 6 months

	ODI within 6 months *n* = 46
Absolute increase in furosemide equivalent dose (mg); Mdn [Q_1_; Q_3_]	10 [10; 40]
1–20 mg; *n* (%)	33 (71.7)
21–40 mg; *n* (%)	7 (15.2)
41–80 mg; *n* (%)	4 (8.7)
> 80 mg, *n* (%)	2 (4.3)

*Mdn* median, *ODI* outpatient diuretic intensification, *Q* quartile

In terms of comorbidities, PNP and lumbar spinal stenosis were significantly more prevalent in patients with ODI. Furthermore, ODI was also associated with higher levels of cardiac biomarkers. Patients with ODI showed a higher IVSd and a higher *E*/*e*’ compared to those without ODI. There were no differences between the two groups regarding specific ATTR-CM treatment.

The independent predictors of ODI in multivariate analysis were higher NYHA class and PNP (Tab. 3). Although 6-MWD was included in the final model, its *p* value missed statistical significance.

**Table 3:** Univariate and multivariate logistic regression with odds ratio for ODI

Variable	Univariate			Multivariate		
	Odds ratio	95% confidence interval	*p*	Odds Ratio	95% confidence interval	*p*
Heart rate	1.026	1.001–1.051	0.038			
NYHA class	2.414	1.353–4.307	0.003	7.496	1.339–41.956	0.022
Lumbar spinal stenosis	3.727	1.578–8.803	0.003			
PNP	2.692	1.305–5.556	0.007	16.635	2.432–113.764	0.004
ln NTproBNP	1.899	1.269–2.843	0.002			
ln Troponin T	2.802	1.238–6.341	0.013			
IVSd	1.118	0.991–1.260	0.070			
E/e’	1.067	1.006–1.131	0.031			
6-MWD	0.995	0.991–0.999	0.011	0.992	0.983–1.001	0.074

*6-MWD* six-minute walk distance, *IVSd* interventricular septal thickness in diastole, *ln* natural logarithm, *NTproBNP* N-terminal pro-brain natriuretic peptide, *NYHA* New York Heart Association, *PNP* polyneuropathy

### Clinical outcomes

The overall estimated 1-year survival and survival free of HF hospitalization was 92.3% (Online Resource 2) and 95.6% (Online Resource 3). Of the 23 patients who experienced HF hospitalization, 52% (12/23) had ODI within the first 6 months. Among the 48% (11/23) without ODI, only one patient had not received any diuretics at baseline.

**Fig. 1:**
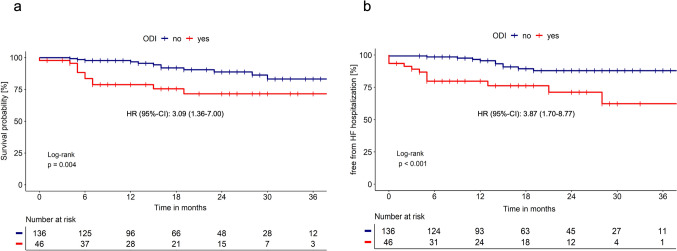
Survival (**a**) and freedom from hospitalization due to HF (**b**) in patients with ATTR-CM, subgrouped by the presence of ODI within the first 6 months after initial presentation

The estimated 2-year survival rate for patients with ODI was 72%, which was significantly lower than 89% for patients without ODI (*p* = 0.004; Fig. 1a). In unadjusted analysis, ODI was associated with a 3.09-fold (95% CI 1.36–7.00; *p* = 0.007) higher risk of death and a 3.87-fold (95% CI 1.70–8.77; *p* = 0.001; Fig. 1b) higher risk of HF hospitalization compared to patients without ODI. When adjusting for NYHA class and UK-NAC stage, the risk still remained 2.38-fold (95% CI 1.03–5.53; *p* = 0.043) and 3.27-fold (95% CI 1.41–7.60; *p* = 0.006) higher, respectively.

**Fig. 2:**
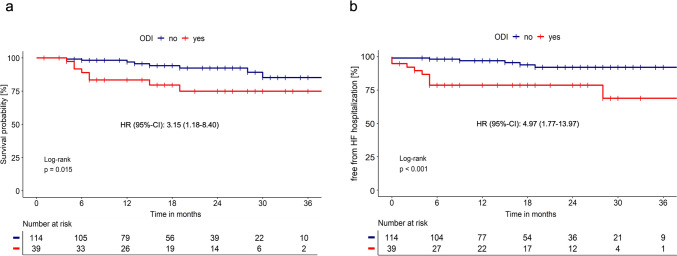
Survival (**a**) and freedom from hospitalization due to HF (**b**) in patients with ATTR-CM treated with tafamidis at baseline, subgrouped by the presence of ODI within the first 6 months after initial presentation

The significant differences in survival (2-year survival: 92% vs. 75%; Fig. 2a) and HF hospitalization (2-year HF hospitalization rate: 7.9% vs. 21.3%; Fig. 2b) persisted when only patients with tafamidis treatment at baseline were analyzed.

**Fig. 3:**
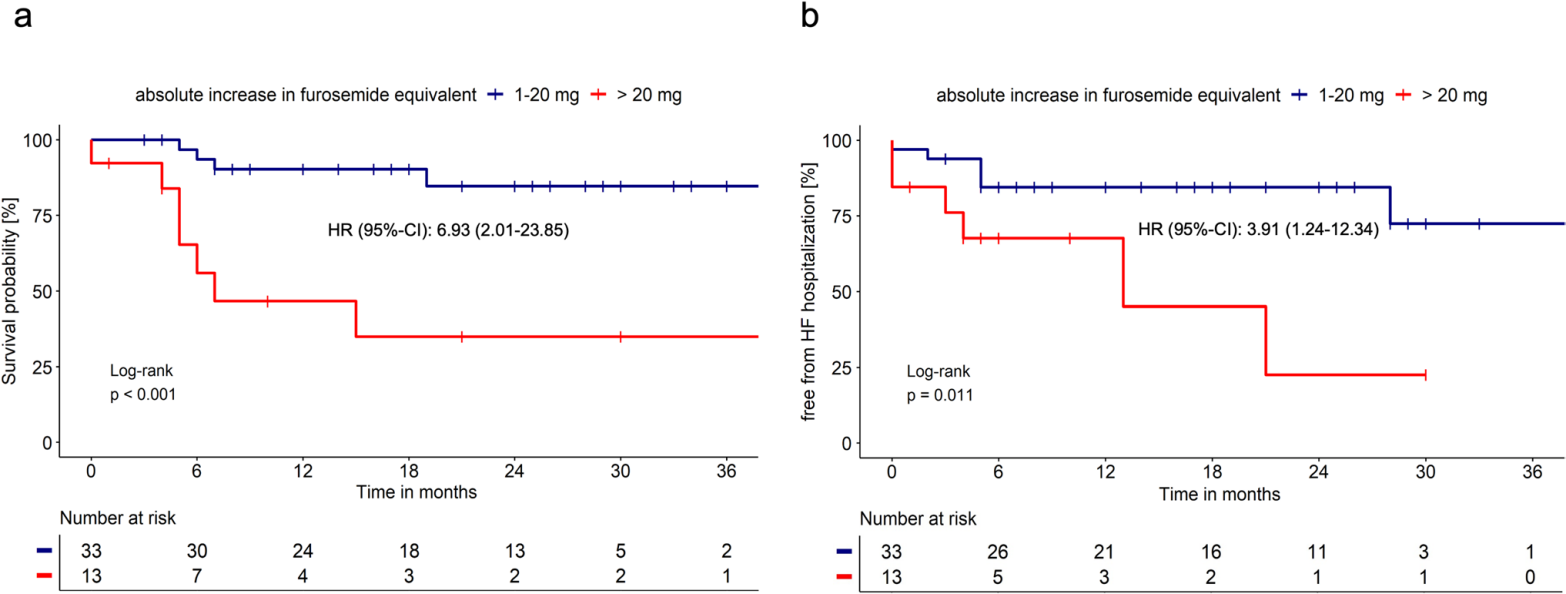
Survival (**a**) and freedom from hospitalization due to HF (**b**) in patients with ATTR-CM experiencing ODI within the first 6 months after initial presentation, stratified by the absolute increase of loop diuretics dose

Among patients with ODI, those who experienced an absolute increase in loop diuretic dose of more than 20 mg of furosemide equivalent had a significantly poorer prognosis compared to those with a lower dose increase (2-year survival: 35% vs. 85% and 2-year HF hospitalization rate: 77.4% vs. 15.5%; Fig. 3).

**Fig. 4:**
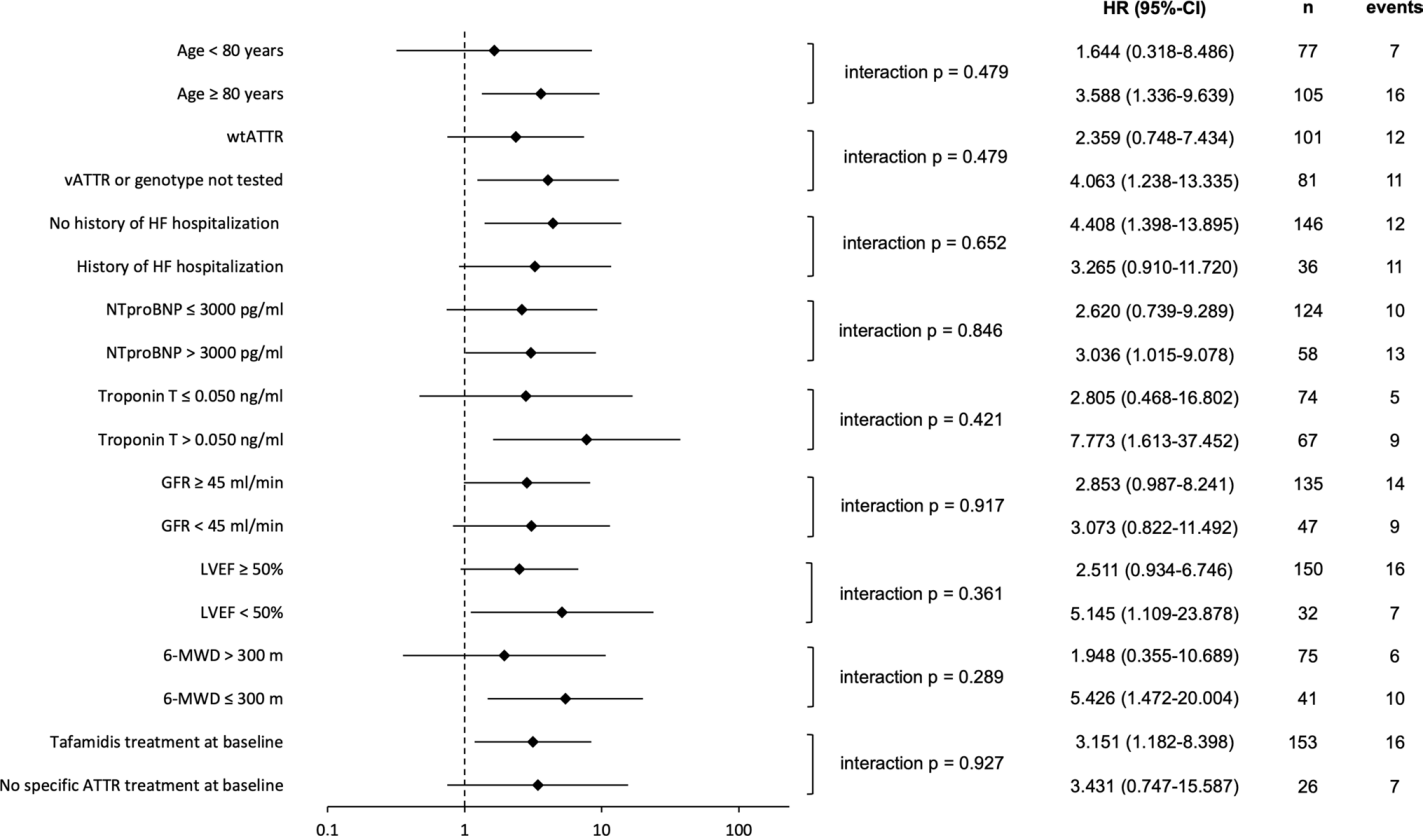
Forest plots regarding all-cause mortality based on the occurrence of ODI within the first 6 months after initial presentation for each clinical subgroup

ODI was associated with an increased hazard of death in important subgroups stratified by clinical and HF risk markers (Fig. 4). There were no significant interactions for the association between ODI and mortality by subgroups.

## Discussion

The main findings of this study are:

- i) ODI occurred in one quarter of the patients within an observational period of six months.
- ii) ODI was associated with advanced ATTR disease with respect to cardiac (NYHA class) and extracardiac amyloidosis manifestation (PNP).
- iii) Both any ODI and the magnitude of the increase in loop diuretic dose were significantly associated with higher all-cause mortality and increased hospitalization rates due to HF.
- iv) The prognostic relevance of ODI was consistent across strata of various clinical and HF risk factors, including age, ATTR subtype, history of cardiac decompensation, cardiac biomarkers, LVEF, 6-MWD, and tafamidis treatment at baseline.

In this study of contemporary patients with ATTR-CM, 25% experienced ODI within a 6-month observational period. This time frame is of clinical relevance since it corresponds to the monitoring interval currently recommended for ATTR-CM patients by experts [6]. The frequency of ODI in our ATTR-CM cohort was clearly higher than that reported for patients with etiologically unselected HF with reduced ejection fraction (HFrEF) (23% during a median follow-up of 12 months [22]) and HF with mildly or preserved ejection fraction (13% to 39% during observational periods of 2.5 and 2.9 years [12, 13]), respectively. This is not unexpected given the progressive nature of ATTR-CM in comparison to overall HF etiologies. Very recently, Ioannou et al. found a 28% and 45% incidence of ODI in two cohorts of ATTR-CM patients over a 12-month observational period [23]. Our slightly higher frequency of ODI per time interval, despite a high rate of specific treatment with tafamidis, might be explained by specific population characteristics, such as older age and a higher proportion of patients with severe symptoms according to NYHA class.

ODI was more frequently observed in patients with baseline features indicative of a more advanced cardiac compromise such as higher NYHA class, lower 6-MWD, elevated cardiac biomarkers and larger wall thickness. Especially, more severe dyspnea and higher NT-proBNP levels in the ODI subgroup are consistent with a hypervolemic volume status, prompting the treating physician to intensify diuretic therapy. Notably, ODI was not associated with a history of hospitalization due to HF, which suggests an additive clinical value of ODI for risk assessment. Furthermore, baseline advanced disease stages (II or III) defined by UK-NAC classification were only numerically higher in patients experiencing ODI, again indicating an additive role of ODI in the assessment of disease severity. The strongest factors associated with ODI in the present analysis were higher NYHA class and PNP. Although extracardiac involvement in form of PNP predominantly occurs in patients with vATTR, Russell et al. [24] reported that PNP is not only common in wtATTR-CM but also associated with a more advanced stage of cardiac disease which might explain our findings. However, there is an ongoing debate about whether extracardiac PNP is caused by ATTR itself or coexisting CTS or lumbar spinal stenosis. In our cohort, we finally cannot exclude a potential overlap between these entities since few patients did not undergo dedicated neurological assessment and PNP diagnosis was obtained from history.

ODI is significantly associated with cardiovascular hospitalizations [13, 22], and cardiovascular and all-cause mortality in patients with HF [12, 13]. Indeed, the incorporation of ODI alongside traditional endpoints in clinical HF trials is currently discussed and highlights the high clinical relevance of this clinical marker [12, 13]. A strong prognostic value for mortality, beyond what is provided by existing risk stratification models, has been demonstrated for oral loop diuretic dose at initial presentation in ATTR-CM patients [25]. We extend these findings in showing that both the intensification and the magnitude of oral loop diuretic dose increases were significantly associated with higher all-cause mortality and increased hospitalization rate due to HF, establishing diuretic dose as a risk marker not only for baseline assessment but also for disease monitoring. Furthermore, our results complement the findings of Zeldin et al. and Ioannou et al., who demonstrated a poorer prognosis for ATTR-CM patients who experience ODI, both in general and within 12 months of observation, respectively [23, 26]. Importantly, in our cohort, ODI remained an independent predictor with a 2.4-fold higher risk of all-cause death and a 3.3-fold higher risk for HF hospitalization after adjusting for NYHA class and UK-NAC stage, which are the mostly used staging systems for disease severity. Current risk stratification models from the UK-NAC and the Mayo Clinic do not consider clinical parameters and focus solely on biomarkers [7, 16]. Given the prognostic significance of ODI and, as previously reported, the initial NYHA class in ATTR-CM patients, incorporating these clinical parameters into risk stratification models may enhance their value [23, 25].

The significant impact of ODI on clinical outcomes persisted even when only patients treated with tafamidis at baseline were analyzed, underscoring its prognostic value in those receiving TTR stabilizers. Considering the 6-month enrollment period during which patients were already on specific ATTR treatment, the slightly higher survival rates in this subgroup may be attributed to the effects of tafamidis, which has demonstrated benefits on all-cause mortality after 18 months [27]. In addition, the prognostic value of ODI remained consistent across various strata, including ages, ATTR subtypes, history of cardiac decompensation, biomarkers used for risk stratification, LVEF, and 6-MWD.

So far, there is limited evidence on optimal monitoring parameters and follow-up intervals for patients with ATTR-CM, although expert consensus has been provided [6]. Suggested monitoring tools include clinical factors, biomarkers, and imaging parameters. In addition to HF hospitalization, which has a well-known impact on prognosis, this study highlights the clinical value of assessing ODI and the absolute increase in diuretic dosage over a short 6-month period [6, 28]. Given the rising prevalence of ATTR-CM and resource constraints, ODI is a valuable, cost-neutral, and simple clinical parameter that can enhance risk stratification for ATTR-CM patients.

### Study limitations

Limitations of this study include its retrospective design and reliance on a single-center cohort. The results are primarily valid for individuals with wtATTR-CM, as the prevalence of vATTR-CM in our cohort was low and approximately one-third of the patients lacked data on ATTR genotype. However, considering the age distribution of these patients, vATTR is estimated to account for only about 6% (~ 4/68) of cases, which is unlikely to significantly alter our results [29]. Regarding ODI, the potential prognostic role of changes in non-loop diuretics, such as thiazides, MRAs, or SGLT2is, and their interaction with loop diuretics, was not assessed. Consideration should be given to the unequal group sizes in some of the performed sub-analyses. A definitive conclusion on ODI and clinical outcomes for patients with low NYHA class or very early disease stages could not be drawn due to the low number of events in these groups. Furthermore, ODI is per definition unsuitable to detect disease progression during the very early stage of ATTR-CM without signs of congestion which might be of increasing clinical relevance in upcoming years due to early detection of patients. Finally, extensive adjusted analysis was not possible to the overall limited number of endpoints. However, it was not the aim to establish ODI as a highly independent predictor within multi-marker risk score. Contrarily, the advantage of ODI is the ready availability in clinical practice which makes its application very likely in contrast to other rather time- and resource-consuming parameters.

## Conclusion

ODI is a common, readily available clinical parameter associated with an increased risk of HF hospitalization and mortality, independent of established clinical risk factors such as NYHA class and UK-NAC stage. These findings provide evidence in support of the integration of ODI in the 6 months monitoring assessments of ATTR-CM patients.

## Supplementary Information

Below is the link to the electronic supplementary material.

Supplementary file1 (PDF 141 KB)

Supplementary file2 (PDF 153 KB)

Supplementary file3 (PDF 165 KB)

## Data Availability

The data from this study are available from the corresponding author upon reasonable request.

## References

[1] Antonopoulos AS, Panagiotopoulos I, Kouroutzoglou A, Koutsis G, Toskas P, Lazaros G, Toutouzas K, Tousoulis D, Tsioufis K, Vlachopoulos C (2022) Prevalence and clinical outcomes of transthyretin amyloidosis: a systematic review and meta-analysis. Eur J Heart Fail 24(9):1677–1696. 10.1002/ejhf.2589 35730461 10.1002/ejhf.2589

[2] Castaño A, Narotsky DL, Hamid N, Khalique OK, Morgenstern R, DeLuca A, Rubin J, Chiuzan C, Nazif T, Vahl T, George I, Kodali S, Leon MB, Hahn R, Bokhari S, Maurer MS (2017) Unveiling transthyretin cardiac amyloidosis and its predictors among elderly patients with severe aortic stenosis undergoing transcatheter aortic valve replacement. Eur Heart J 38(38):2879–2887. 10.1093/eurheartj/ehx350 29019612 10.1093/eurheartj/ehx350 PMC5837725

[3] Hahn VS, Yanek LR, Vaishnav J, Ying W, Vaidya D, Lee YZJ, Riley SJ, Subramanya V, Brown EE, Hopkins CD, Ononogbu S, Perzel Mandell K, Halushka MK, Steenbergen C Jr, Rosenberg AZ, Tedford RJ, Judge DP, Shah SJ, Russell SD, Kass DA, Sharma K (2020) Endomyocardial biopsy characterization of heart failure with preserved ejection fraction and prevalence of cardiac amyloidosis. JACC Heart Fail 8(9):712–724. 10.1016/j.jchf.2020.04.007 32653448 10.1016/j.jchf.2020.04.007 PMC7604801

[4] Maurer MS, Schwartz JH, Gundapaneni B, Elliott PM, Merlini G, Waddington-Cruz M, Kristen AV, Grogan M, Witteles R, Damy T, Drachman BM, Shah SJ, Hanna M, Judge DP, Barsdorf AI, Huber P, Patterson TA, Riley S, Schumacher J, Stewart M, Sultan MB, Rapezzi C (2018) Tafamidis treatment for patients with transthyretin amyloid cardiomyopathy. N Engl J Med 379(11):1007–1016. 10.1056/NEJMoa1805689 30145929 10.1056/NEJMoa1805689

[5] Fontana M, Berk JL, Gillmore JD, Witteles RM, Grogan M, Drachman B, Damy T, Garcia-Pavia P, Taubel J, Solomon SD, Sheikh FH, Tahara N, Gonzalez-Costello J, Tsujita K, Morbach C, Pozsonyi Z, Petrie MC, Delgado D, Van der Meer P, Jabbour A, Bondue A, Kim D, Azevedo O, Hvitfeldt Poulsen S, Yilmaz A, Jankowska EA, Algalarrondo V, Slugg A, Garg PP, Boyle KL, Yureneva E, Silliman N, Yang L, Chen J, Eraly SA, Vest J, Maurer MS, Investigators H-BT (2024) Vutrisiran in patients with transthyretin amyloidosis with cardiomyopathy. N Engl J Med. 10.1056/NEJMoa240913439555828

[6] Garcia-Pavia P, Bengel F, Brito D, Damy T, Duca F, Dorbala S, Nativi-Nicolau J, Obici L, Rapezzi C, Sekijima Y, Elliott PM (2021) Expert consensus on the monitoring of transthyretin amyloid cardiomyopathy. Eur J Heart Fail 23(6):895–905. 10.1002/ejhf.2198 33915002 10.1002/ejhf.2198 PMC8239846

[7] Gillmore JD, Damy T, Fontana M, Hutchinson M, Lachmann HJ, Martinez-Naharro A, Quarta CC, Rezk T, Whelan CJ, Gonzalez-Lopez E, Lane T, Gilbertson JA, Rowczenio D, Petrie A, Hawkins PN (2018) A new staging system for cardiac transthyretin amyloidosis. Eur Heart J 39(30):2799–2806. 10.1093/eurheartj/ehx589 29048471 10.1093/eurheartj/ehx589

[8] Ney S, Gertz RJ, Pennig L, Nies RJ, Holtick U, Volker LA, Wunderlich G, Seuthe K, Hohmann C, Metze C, Nahle CP, von Stein J, Bruwer M, Ten Freyhaus H, Pfister R (2024) Multiparametric monitoring of disease progression in contemporary patients with wild-type transthyretin amyloid cardiomyopathy initiating tafamidis treatment. J Clin Med. 10.3390/jcm13010284 38202291 10.3390/jcm13010284 PMC10779991

[9] Vergaro G, Gentile F, Meems LMG, Aimo A, Januzzi JL Jr, Richards AM, Lam CSP, Latini R, Staszewsky L, Anand IS, Cohn JN, Ueland T, Gullestad L, Aukrust P, Brunner-La Rocca HP, Bayes-Genis A, Lupon J, Yoshihisa A, Takeishi Y, Egstrup M, Gustafsson I, Gaggin HK, Eggers KM, Huber K, Gamble GD, Ling LH, Leong KTG, Yeo PSD, Ong HY, Jaufeerally F, Ng TP, Troughton R, Doughty RN, Devlin G, Lund M, Giannoni A, Passino C, de Boer RA, Emdin M (2021) NT-proBNP for risk prediction in heart failure: identification of optimal cutoffs across body mass index categories. JACC Heart Fail 9(9):653–663. 10.1016/j.jchf.2021.05.014 34246607 10.1016/j.jchf.2021.05.014

[10] Madamanchi C, Alhosaini H, Sumida A, Runge MS (2014) Obesity and natriuretic peptides, BNP and NT-proBNP: mechanisms and diagnostic implications for heart failure. Int J Cardiol 176(3):611–617. 10.1016/j.ijcard.2014.08.007 25156856 10.1016/j.ijcard.2014.08.007 PMC4201035

[11] Metra M, Tomasoni D, Adamo M, Bayes-Genis A, Filippatos G, Abdelhamid M, Adamopoulos S, Anker SD, Antohi L, Bohm M, Braunschweig F, Gal TB, Butler J, Cleland JGF, Cohen-Solal A, Damman K, Gustafsson F, Hill L, Jankowska EA, Lainscak M, Lund LH, McDonagh T, Mebazaa A, Moura B, Mullens W, Piepoli M, Ponikowski P, Rakisheva A, Ristic A, Savarese G, Seferovic P, Sharma R, Tocchetti CG, Yilmaz MB, Vitale C, Volterrani M, von Haehling S, Chioncel O, Coats AJS, Rosano G (2023) Worsening of chronic heart failure: definition, epidemiology, management and prevention. A clinical consensus statement by the Heart Failure Association of the European Society of Cardiology. Eur J Heart Fail 25(6):776–791. 10.1002/ejhf.2874 37208936 10.1002/ejhf.2874

[12] Chatur S, Vaduganathan M, Claggett BL, Cunningham JW, Docherty KF, Desai AS, Jhund PS, de Boer RA, Hernandez AF, Inzucchi SE, Kosiborod MN, Lam CSP, Martinez FA, Shah SJ, Petersson M, Langkilde AM, McMurray JJV, Solomon SD (2023) Outpatient worsening among patients with mildly reduced and preserved ejection fraction heart failure in the DELIVER trial. Circulation 148(22):1735–1745. 10.1161/CIRCULATIONAHA.123.066506 37632455 10.1161/CIRCULATIONAHA.123.066506 PMC10664793

[13] Ferreira JP, Liu J, Claggett BL, Vardeny O, Pitt B, Pfeffer MA, Solomon SD, Zannad F (2022) Outpatient diuretic intensification as endpoint in heart failure with preserved ejection fraction trials: an analysis from TOPCAT. Eur J Heart Fail 24(2):378–384. 10.1002/ejhf.2376 34755426 10.1002/ejhf.2376

[14] Arbelo E, Protonotarios A, Gimeno JR, Arbustini E, Barriales-Villa R, Basso C, Bezzina CR, Biagini E, Blom NA, de Boer RA, De Winter T, Elliott PM, Flather M, Garcia-Pavia P, Haugaa KH, Ingles J, Jurcut RO, Klaassen S, Limongelli G, Loeys B, Mogensen J, Olivotto I, Pantazis A, Sharma S, Van Tintelen JP, Ware JS, Kaski JP, Group ESCSD (2023) 2023 ESC Guidelines for the management of cardiomyopathies. Eur Heart J 44(37):3503–3626. 10.1093/eurheartj/ehad194 37622657 10.1093/eurheartj/ehad194

[15] Garcia-Pavia P, Rapezzi C, Adler Y, Arad M, Basso C, Brucato A, Burazor I, Caforio ALP, Damy T, Eriksson U, Fontana M, Gillmore JD, Gonzalez-Lopez E, Grogan M, Heymans S, Imazio M, Kindermann I, Kristen AV, Maurer MS, Merlini G, Pantazis A, Pankuweit S, Rigopoulos AG, Linhart A (2021) Diagnosis and treatment of cardiac amyloidosis: a position statement of the ESC Working Group on Myocardial and Pericardial Diseases. Eur Heart J 42(16):1554–1568. 10.1093/eurheartj/ehab072 33825853 10.1093/eurheartj/ehab072 PMC8060056

[16] Grogan M, Scott CG, Kyle RA, Zeldenrust SR, Gertz MA, Lin G, Klarich KW, Miller WL, Maleszewski JJ, Dispenzieri A (2016) Natural history of wild-type transthyretin cardiac amyloidosis and risk stratification using a novel staging system. J Am Coll Cardiol 68(10):1014–1020. 10.1016/j.jacc.2016.06.033 27585505 10.1016/j.jacc.2016.06.033

[17] Lavine SJ, Murtaza G, Rahman ZU, Kelvas D, Paul TK (2021) Diastolic function grading by American Society of Echocardiography guidelines and prediction of heart failure readmission and all-cause mortality in a community-based cohort. Echocardiography 38(12):1988–1998. 10.1111/echo.15206 34555216 10.1111/echo.15206

[18] Lang RM, Bierig M, Devereux RB, Flachskampf FA, Foster E, Pellikka PA, Picard MH, Roman MJ, Seward J, Shanewise JS, Solomon SD, Spencer KT, Sutton MS, Stewart WJ, Chamber Quantification Writing Group, American Society of Echocardiography’s Guidelines and Standards Committee, European Association of Echocardiography (2005) Recommendations for chamber quantification: a report from the American Society of Echocardiography’s Guidelines and Standards Committee and the Chamber Quantification Writing Group, developed in conjunction with the European Association of Echocardiography, a branch of the European Society of Cardiology. J Am Soc Echocardiogr 18(12):1440–1463. 10.1016/j.echo.2005.10.005 16376782 10.1016/j.echo.2005.10.005

[19] Afilalo J, Lauck S, Kim DH, Lefevre T, Piazza N, Lachapelle K, Martucci G, Lamy A, Labinaz M, Peterson MD, Arora RC, Noiseux N, Rassi A, Palacios IF, Genereux P, Lindman BR, Asgar AW, Kim CA, Trnkus A, Morais JA, Langlois Y, Rudski LG, Morin JF, Popma JJ, Webb JG, Perrault LP (2017) Frailty in older adults undergoing aortic valve replacement: the FRAILTY-AVR study. J Am Coll Cardiol 70(6):689–700. 10.1016/j.jacc.2017.06.024 28693934 10.1016/j.jacc.2017.06.024

[20] Ioannou A, Fumagalli C, Razvi Y, Porcari A, Rauf MU, Martinez-Naharro A, Venneri L, Moody W, Steeds RP, Petrie A, Whelan C, Wechalekar A, Lachmann H, Hawkins PN, Solomon SD, Gillmore JD, Fontana M (2024) Prognostic value of a 6-minute walk test in patients with transthyretin cardiac amyloidosis. J Am Coll Cardiol. 10.1016/j.jacc.2024.04.011 38739065 10.1016/j.jacc.2024.04.011 PMC11218050

[21] Mentz RJ, Anstrom KJ, Eisenstein EL, Sapp S, Greene SJ, Morgan S, Testani JM, Harrington AH, Sachdev V, Ketema F, Kim DY, Desvigne-Nickens P, Pitt B, Velazquez EJ, Investigators T-H (2023) Effect of torsemide vs furosemide after discharge on all-cause mortality in patients hospitalized with heart failure: the TRANSFORM-HF randomized clinical trial. JAMA 329(3):214–223. 10.1001/jama.2022.23924 36648467 10.1001/jama.2022.23924 PMC9857435

[22] Khan MS, Greene SJ, Hellkamp AS, DeVore AD, Shen X, Albert NM, Patterson JH, Spertus JA, Thomas LE, Williams FB, Hernandez AF, Fonarow GC, Butler J (2021) Diuretic changes, health care resource utilization, and clinical outcomes for heart failure with reduced ejection fraction: from the change the management of patients with heart failure registry. Circ Heart Fail 14(11):e008351. 10.1161/CIRCHEARTFAILURE.121.008351 34674536 10.1161/CIRCHEARTFAILURE.121.008351

[23] Ioannou A, Cappelli F, Emdin M, Nitsche C, Longhi S, Masri A, Cipriani A, Zampieri M, Colio F, Poledniczek M, Porcari A, Razvi Y, Aimo A, Vergaro G, De Michieli L, Rauf MU, Patel RK, Villanueva E, Lustig Y, Venneri L, Martinez-Naharro A, Lachmann H, Wechalekar A, Whelan C, Petrie A, Hawkins PN, Solomon S, Gillmore JD, Fontana M (2024) stratifying disease progression in patients with cardiac ATTR amyloidosis. J Am Coll Cardiol 83(14):1276–1291. 10.1016/j.jacc.2023.12.036 38530684 10.1016/j.jacc.2023.12.036 PMC11004588

[24] Russell A, Hahn C, Chhibber S, Korngut L, Fine NM (2021) Utility of neuropathy screening for wild-type transthyretin amyloidosis patients. Can J Neurol Sci 48(5):607–615. 10.1017/cjn.2020.271 33342448 10.1017/cjn.2020.271

[25] Cheng RK, Levy WC, Vasbinder A, Teruya S, De Los SJ, Leedy D, Maurer MS (2020) Diuretic dose and NYHA functional class are independent predictors of mortality in patients with transthyretin cardiac amyloidosis. JACC CardioOncol 2(3):414–424. 10.1016/j.jaccao.2020.06.007 33073249 10.1016/j.jaccao.2020.06.007 PMC7561022

[26] Zeldin L, Eichler JBS, Teruya SL, Weinsaft AY, Mirabal A, Cuomo MO, Mateo K, Helmke S, Maurer MS (2024) Outpatient worsening of heart failure and mortality in transthyretin amyloid cardiomyopathy. Eur J Heart Fail. 10.1002/ejhf.3540 39638976 10.1002/ejhf.3540

[27] Maurer MS, Schwartz JH, Gundapaneni B, Elliott PM, Merlini G, Waddington-Cruz M, Kristen AV, Grogan M, Witteles R, Damy T, Drachman BM, Shah SJ, Hanna M, Judge DP, Barsdorf AI, Huber P, Patterson TA, Riley S, Schumacher J, Stewart M, Sultan MB, Rapezzi C, Investigators A-AS (2018) Tafamidis treatment for patients with transthyretin amyloid cardiomyopathy. N Engl J Med 379(11):1007–1016. 10.1056/NEJMoa1805689 30145929 10.1056/NEJMoa1805689

[28] Ladefoged BT, Dybro A, Dahl Pedersen AL, Rasmussen TB, Vase HO, Clemmensen TS, Gillmore J, Poulsen SH (2022) Incidence and predictors of worsening heart failure in patients with wild-type transthyretin cardiac amyloidosis. ESC Heart Fail 9(5):2978–2987. 10.1002/ehf2.14000 35733407 10.1002/ehf2.14000 PMC9715879

[29] Maestro-Benedicto A, Vela P, de Frutos F, Mora N, Pomares A, Gonzalez-Vioque E, Briceno A, Cabrera E, Cobo-Marcos M, Dominguez F, Gonzalez-Lopez E, Segovia J, Lara-Pezzi E, Garcia-Pavia P (2022) Frequency of hereditary transthyretin amyloidosis among elderly patients with transthyretin cardiomyopathy. Eur J Heart Fail 24(12):2367–2373. 10.1002/ejhf.2658 35999650 10.1002/ejhf.2658 PMC10087903

