# Supplementary material for: Outpatient diuretic intensification: a simple prognostic marker in cardiac transthyretin amyloidosis
Source: Clin Res Cardiol. 2025 Mar 4;115(5):752–62. doi: 10.1007/s00392-025-02617-4 (PMC13083326; doi:10.1007/s00392-025-02617-4)
Supplement: Supplementary file 1 — Supplementary file1 (PDF 141 KB) [file 392_2025_2617_MOESM1_ESM.pdf]

Outpatient Diuretic Intensification: a Simple Prognostic Marker in Cardiac Transthyretin Amyloidosis

Clinical Research in Cardiology

- Online Resource 1 -

Richard J. Nies[1], Svenja Ney[1], Jasper F. Nies[2], Katharina Seuthe[1], Lukas Klösges[1], Monique Brüwer[1], Stephan Nienaber[1], Sascha Macherey-Meyer[1], Matthieu Schäfer[1], Roman Pfister[1]

[1] University of Cologne, Faculty of Medicine and University Hospital Cologne, Clinic III for Internal Medicine, Kerpener Str. 62, 50937 Cologne

[2] Department of Nephrology, University of Cologne, Kerpener Str. 62, D-50937 Cologne, Germany

Corresponding author:

Dr. med. Richard Nies

University of Cologne, Faculty of Medicine and University Hospital Cologne

Clinic III for Internal Medicine

Kerpener Straße 62

D-50937 Köln, Germany

Phone: +49 221 47876653

Fax: +49 221 47832343

E-mail: richard.nies@uk-koeln.de

Online Resource 1 Flowchart regarding patient selection

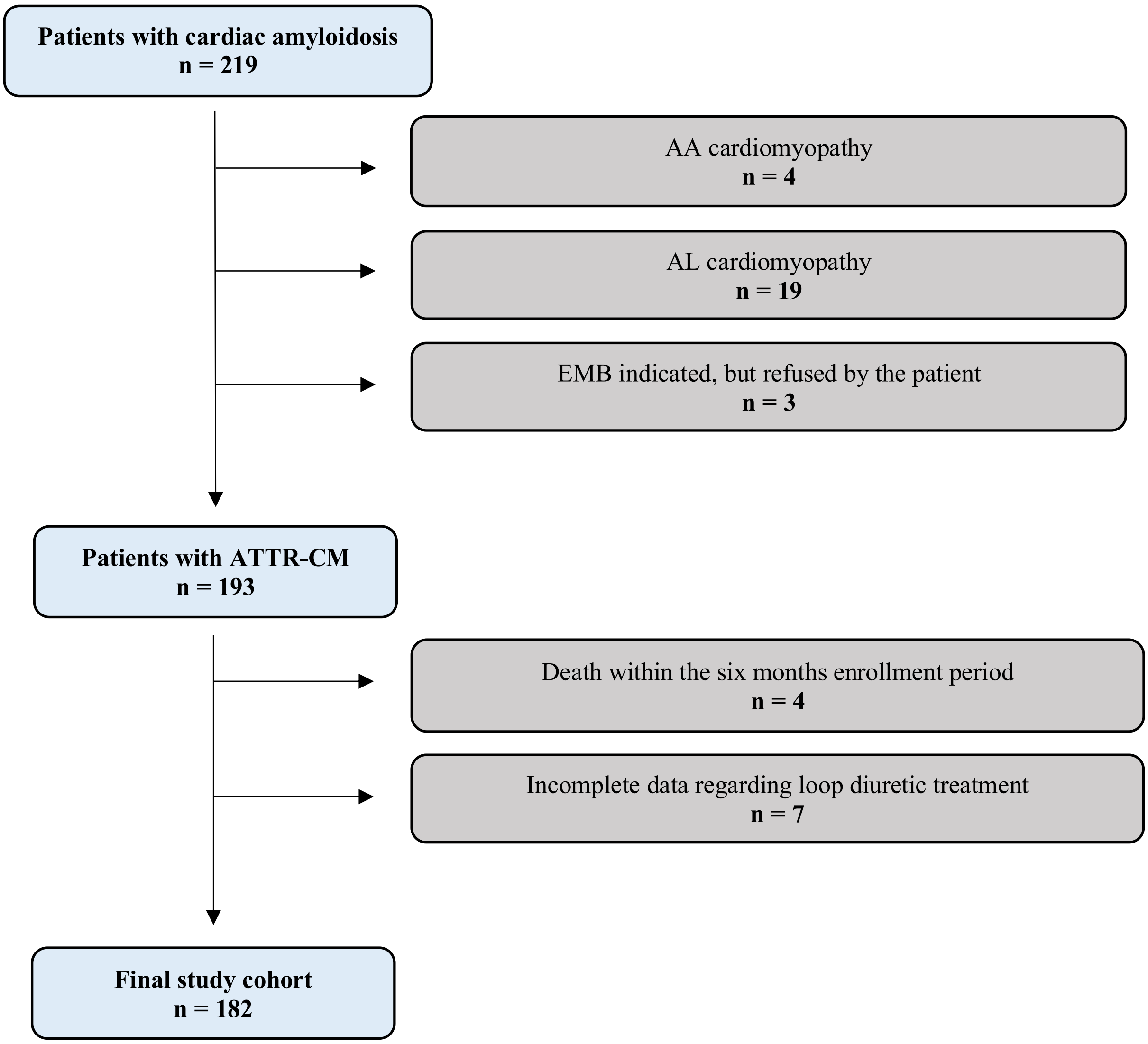

AA = amyloid A; AL = amyloid light-chain; ATTR-CM = transthyretin amyloidosis cardiomyopathy; EMB = endomyocardial biopsy.
